# “Single Coronary Artery” from Right Sinus—Uncommon Causes of Ischemia with Non-Obstructive Coronary Arteries

**DOI:** 10.3390/diagnostics15151971

**Published:** 2025-08-06

**Authors:** Paweł Muszyński, Marlena Święcicka, Dominika Musiałowska, Dorota Pura, Małgorzata Kazberuk, Anna Kożuchowska-Eljasiewicz, Caroline Sasinowski, Urszula Bajda, Wiktoria Grądzka-Matys, Anna Tomaszuk-Kazberuk

**Affiliations:** 1 Department of Cardiology, Lipidology and Internal Diseases, Medical University of Bialystok, Żurawia 14, 15-569 Bialystok, Poland; 2 Faculty of Medicine, Medical University of Bialystok, Kilińskiego 1, 15-089 Białystok, Poland

**Keywords:** coronary artery, coronary artery anomalies, computer tomography

## Abstract

Anomalies of coronary artery origins are rare but significant conditions that can range from benign to life-threatening. Early detection through imaging is crucial in preventing adverse outcomes. The treatment strategy varies depending on the type and severity of the anomaly, ranging from pharmacological treatment to surgery. A 22-year-old male patient, after syncope, after excluding other causes, had an exercise drill test, which was clinically negative and ECG-positive. Angio-CT revealed an undeveloped left main coronary artery (LMCA), and the circulation was supplied through the right coronary artery (RCA). The RCA provides the left anterior descending artery (LAD), and the LAD retrogradely supplies the left circumflex artery (LCX). The myocardial perfusion scintigraphy showed a slight lack of perfusion in the anterior wall (6% of total perfusion). The patient was qualified for further observation. A 77-year-old female underwent cardiac CT due to stenocardia. CT showed a lack of LMCA. The initial segment of the RCA gave rise to the left coronary artery (LCA), which encircled the aortic bulb posteriorly and bifurcated into branches resembling the LCX and LAD. After the Heart Team consultation, the patient was deemed eligible for conservative treatment. Angio-CT is a valuable tool for detecting coronary artery anomalies.

**Figure 1:**
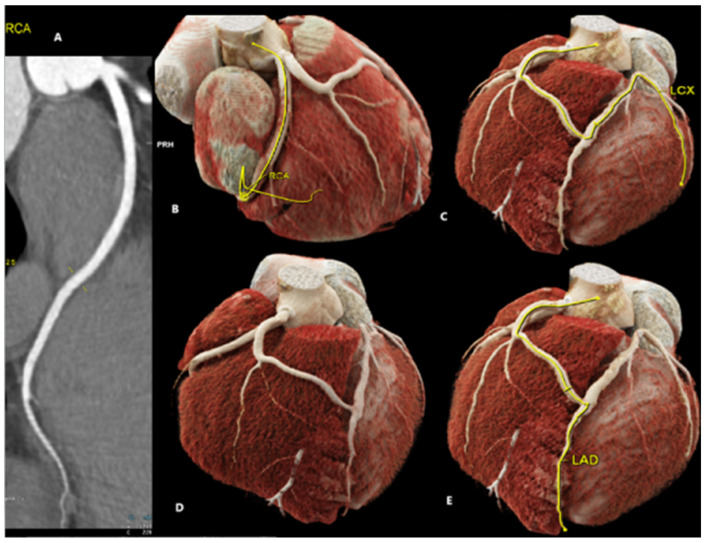
A 22-year-old male patient with a history of hypertension, right bundle branch block (RBBB), and asthma was admitted to the Cardiac Unit following an episode of syncope preceded by symptoms of shortness of breath and temporal visual disturbances. The patient reported experiencing previous episodes of syncope before his 16th birthday but had remained asymptomatic in the past eight years. Electrocardiography (ECG) showed sinus rhythm (64 bpm), right bundle branch block (RBBB), left ventricular hypertrophy, and features of early repolarization. The diagnosis of hypertension was confirmed by ambulatory blood pressure monitoring (ABPM). Imaging studies, including chest X-ray, abdominal ultrasound, carotid Doppler ultrasound, and echocardiography were unremarkable. Twenty-four-hour Holter monitoring recorded sinus rhythm with infrequent ventricular and supraventricular arrhythmias (<1% of the recording). An exercise stress test was clinically negative and ECG-positive with no rhythm or conduction disturbances. The orthostatic test was also negative. Subsequently, an outpatient coronary artery angio-CT was performed, revealing an undeveloped left main coronary artery (LMCA). Angio-CT showing RCA (**A**) and 3D models (**B**–**E**) are presented in Figure 1.

**Figure 2:**
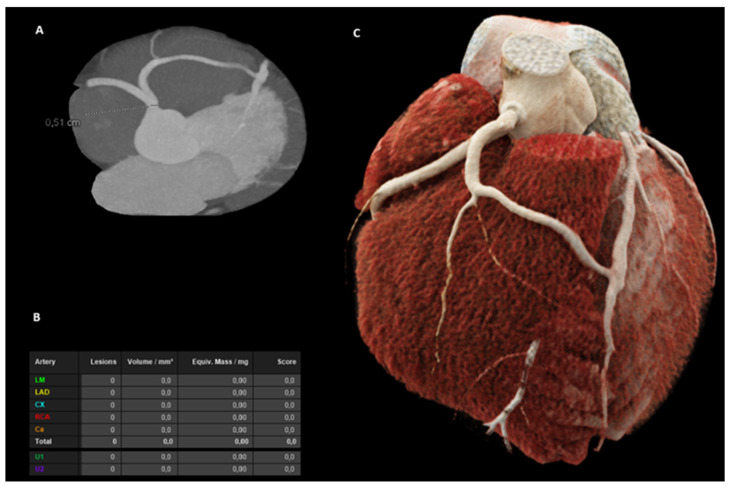
Coronary circulation was supplied via the right coronary artery (RCA), which arose normally from the aorta. The left anterior descending artery (LAD) originated from a wide branch of the RCA, while the LAD retrogradely supplied the left circumflex artery (LCX). After the Heart Team consultation, the patient was deemed qualified for coronary artery bypass grafting in the event of evidence of significant ischemia on single-photon emission computed tomography (SPECT). (**A**) Angio-CT visualization, (**B**) calcium score, and (**C**) 3D presentation.

**Figure 3:**
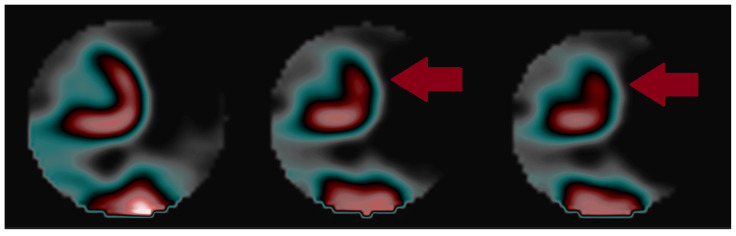
Performed SPECT showed slightly decreased flow at rest in the subapical segment (13), and at exercise after achieving 8.6 METs, a decrease in perfusion in a segment of the anterior wall (1 and 7) with a total flow decrease of 6% (mild reduction). The patient qualified for conservative treatment and observation.

**Figure 4:**
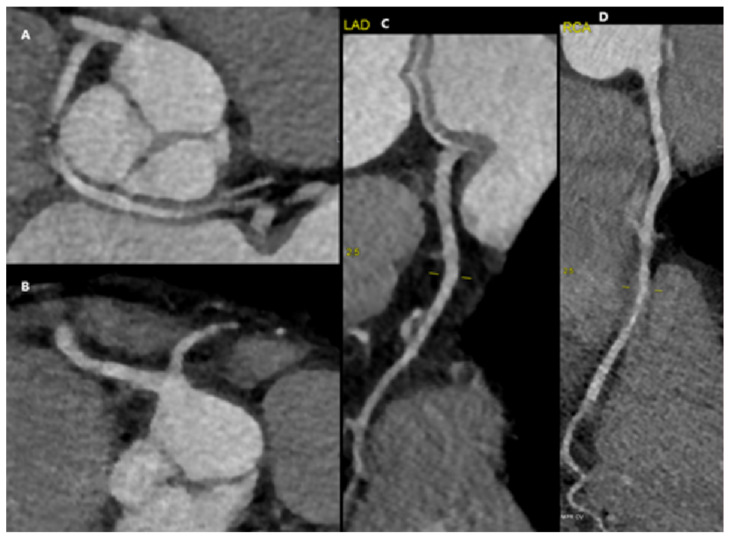
A 77-year-old female complaining of a typical chest pain (retrosternal chest discomfort provoked by exertion relieved by rest) was admitted to the hospital. The resting 12-lead electrocardiography (ECG) showed sinus rhythm at 69 bpm, without repolarization abnormalities. The estimated Risk-Factor-Weighted Clinical Likelihood (RF-CL) of obstructive CAD was moderate (23%). The echocardiography did not detect regional wall motion abnormalities, and the left ventricular ejection fraction (LVEF) was 60%, without valvular heart disease. Carotid Doppler ultrasound showed atherosclerotic changes without significant hemodynamic obstructiveness. A chest X-ray ruled out other potential causes of chest pain. The patient was qualified for coronary artery angio-CT, which showed an anomaly of the origin of the left coronary artery—two main branches of the left coronary artery were arising from the proximal segment of the right coronary artery. (**A**,**B**) Presentation of branches arising from the proximal RCA trunk. (**C**,**D**) Angio-CT showing non-obstructive LAD and RCA.

**Figure 5:**
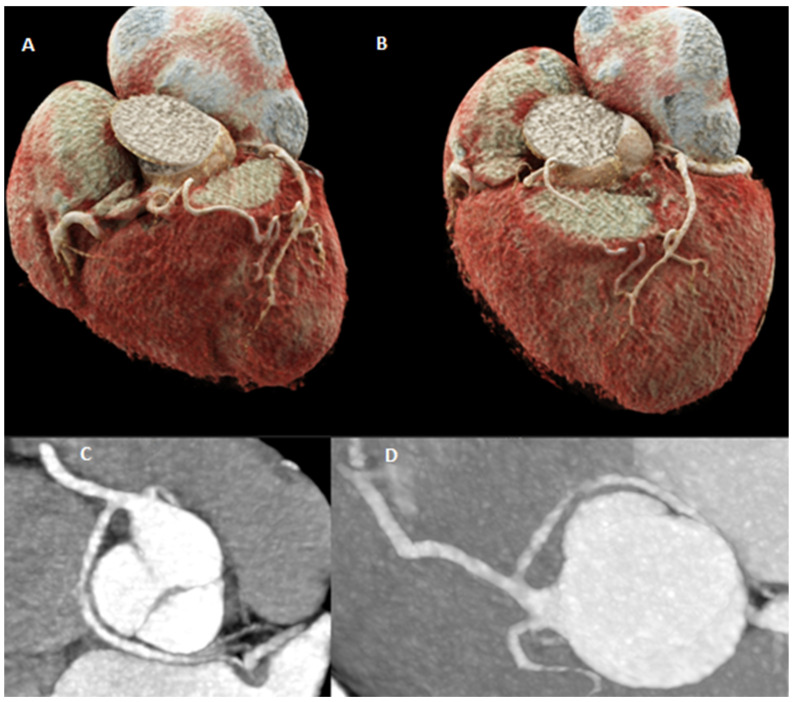
The first one runs backward from the aortic root and then bifurcates into 3 small vessels acting as the circumflex and obtuse marginal arteries. The second one is divided into two branches—one well-developed septal branch and the second runs forward as a tortuous vessel from the arterial cone and heads typically towards the anterior interventricular sulcus. Coronary arteries do not have obstruction. After diagnosis, the patient had exercise ECG to complement her clinical evaluation for assessing symptoms, ST-segment changes, exercise tolerance, arrhythmias, BP response, and event risk. The exercise stress test (6.5 MET) did not show signs of ischemia in ECG. During the test, numerous extra premature ventricular contractions were observed—the patient felt them as chest pain. The PVCs stopped in the recovery phase. The control forty-eight-hour Holter monitoring recorded sinus rhythm with infrequent ventricular and supraventricular arrhythmias (<1% of the recording—1174 PVCs). For optimal decision-making, the patient was consulted by a Heart Team. A multidisciplinary care team decided on conservative treatment. (**A**,**B**) Three-dimensional visualization; (**C**,**D**) angio-CT presentation. Single coronary artery (SCA) is a rare anomaly, found in 0.024–0.066% of patients undergoing coronary artery catheterization and 0.024 and 0.098% undergoing coronary computed tomography angiography (CTA) [1,2]. The majority of patients remain asymptomatic; rarely, SCA can result in chest pain, myocardial infarction, syncope, ventricular tachycardia, and even sudden cardiac death [1]. “Malignant variants” include an intra-arterial course running between the great vessels [3]. CTA allows for the non-invasive assessment of coronary artery anomaly; however, it may not allow for the assessment of clinical significance and may require an additional stress test for signs of ischemia. In the presence of significant ischemia, the surgical treatment is the preferable option [3]. The conservative treatment includes the use of beta-blockers and restriction of physical activity. The angiographic classification by Lipton et al. divides SCA into Group 1 (solitary vessel follows the course of either a normal right or left coronary artery), Group 2 (the single coronary artery arises from the right or left coronary sinus, and from this, a very large trunk crosses the base of the heart to arrive in the vicinity of the normal contralateral coronary artery), and Group 3 (single coronary artery originating from the right sinus; the circumflex branch and the anterior descending branch may arise separately from a common trunk) [4]. Both presented cases can be classified as RII-A type.

## References

[1] Singh N., Gupta Y., Singh B., Agrawal G.R., Rajput S. (2022). Diagnosis and demonstration of single coronary artery by multidetector CT angiography: Series of two cases. Egypt. J. Radiol. Nucl. Med..

[2] Gąsecka A., Jakubik O., Putowska P., Pietrasik A. (2021). Single coronary artery anomaly. Arch. Med. Sci.—Civiliz. Dis..

[3] Bigler M.R., Kadner A., Räber L., Ashraf A., Windecker S., Siepe M., Padalino M.A., Gräni C. (2022). Therapeutic Management of Anomalous Coronary Arteries Originating from the Opposite Sinus of Valsalva: Current Evidence, Proposed Approach, and the Unknowing. J. Am. Heart Assoc..

[4] Lipton M.J., Barry W.H., Obrez I., Silverman J.F., Wexler L. (1979). Isolated Single Coronary Artery: Diagnosis, Angiographic Classification, and Clinical Significance. Radiology.

